# A Cryptic Case of an Anaerobic Hepatic Abscess Following a Cesarean Section

**DOI:** 10.7759/cureus.37293

**Published:** 2023-04-08

**Authors:** Andrew M Cyr, Marc Perlman, Cassandra L Denefrio, Jessica Kumar, Alan Sanders

**Affiliations:** 1 Department of Internal Medicine, Albany Medical College, Albany, USA; 2 Department of Obstetrics and Gynecology, Albany Medical Center, Albany, USA; 3 Department of Medicine, Division of Infectious Disease, Albany Medical Center, Albany, USA

**Keywords:** finegoldia magna, peptoniphilus ssp, subhepatic liver abscess, periuterine hematoma, chorioamnionitis complications, postpartum abscess

## Abstract

Postpartum infectious complications can present with a wide range of nonspecific symptoms. Herein, we describe a complicated late postpartum presentation of recurrent fever following a cesarean delivery complicated by chorioamnionitis. Following discharge, the patient experienced cyclical fever and was treated with antipyretics as an outpatient. The patient continued to experience symptoms and reported to the hospital for further evaluation. Initially thought to be septic pelvic thrombophlebitis, the patient was trialed on clindamycin and gentamycin without resolution of symptoms. After extensive evaluation, the fevers were found to be the result of an infected periuterine hematoma and a concomitant subcapsular inferior hepatic abscess. Bacterial cultures isolated two rare anaerobic organisms: *Peptoniphilus ssp. *and *Finegoldia magna.* Source control was achieved by drainage of the two abscesses followed by antibiotic treatment with ertapenem and metronidazole, and the patient recovered successfully. This is the first reported case, to the authors' knowledge, of this complicated postpartum picture due to these anaerobic organisms.

## Introduction

Women who deliver by cesarean section experience higher rates of morbidity and mortality than women who deliver vaginally, often due to complications such as endometritis, wound infection, and urinary tract infections [1]. Other common complications include gastrointestinal issues, deep venous thrombosis, and septic pelvic thrombophlebitis. Intrapartum chorioamnionitis increases the risk for cesarean section and subsequent endomyometritis, wound infection, pelvic abscess, bacteremia, and postpartum hemorrhage by two to four-fold [2]. Although prophylactic antibiotics decrease the rate of postoperative infection from as much as 85% to about 5% [3], one study estimates nearly 94% of those infections manifest outside of the hospital following discharge [4]. Herein, we describe a complicated late postpartum presentation of recurrent fevers following cesarean delivery and chorioamnionitis found to be the result of an infected periuterine hematoma and a coincidental subcapsular inferior hepatic abscess with the same unusual anaerobic organisms.

## Case presentation

A 31-year-old Asian female G2P0010 was admitted to labor and delivery for term induction of labor at 40+1 weeks gestational age. Past medical history and family history were noncontributory. On admission, the patient's vitals were stable, and fetal cardiotocography revealed a baseline heart rate of 150 BPM with moderate variability, spontaneous accelerations, and an absence of decelerations. Labor was induced with a single Foley balloon, followed by artificial rupture of membranes and oxytocin. During active labor, the patient became febrile to 40.1°C and tachycardic. Fetal tachycardia was also noted, but no blood cultures were obtained at this time. Complete blood count was within normal limits with a white blood cell count of 8.9 10^3^/μL, hemoglobin 11.7 g/dL, and platelet count of 311 10^3^/μL. She was given three doses of clindamycin 900 mg (due to penicillin allergy) and two doses of gentamicin 280 mg for suspected chorioamnionitis. The patient reached 7.5 cm dilated with the fetus at -2 station and experienced no further cervical change for 13 hours due to inadequate contractions. Fetal monitoring was within normal limits. After 27 hours and 28 minutes of active labor, the patient underwent primary cesarean delivery for the arrest of dilation, which was complicated by postpartum hemorrhage with a blood loss of 1500 mL secondary to uterine atony, which was responsive to bimanual massage and uterotonic medications. Delivery and subsequent hemodynamic stabilization took one hour and nine minutes in total. Postoperatively, she received one unit of packed red blood cells (pRBCs) and her platelets were 247 10^3^/μL. The neonate was born at 4135 g, met all milestones, and was deemed safe for discharge home. The patient met all postpartum milestones and was discharged on postoperative day 3 with no complications.

On postoperative day 10, the patient was seen at the outpatient obstetrics clinic following a fever of 38.3°C that responded to acetaminophen and fatigue. She was breastfeeding without complication. There were no signs of wound infection or mastitis at that time. On postoperative day 11, the patient was again febrile at home and again responsive to acetaminophen. The patient reported to her primary care physician with a fever on postoperative day 12, where a CBC showed significant thrombocytosis, and she was encouraged to present to the hospital for further evaluation.

**Figure 1:**
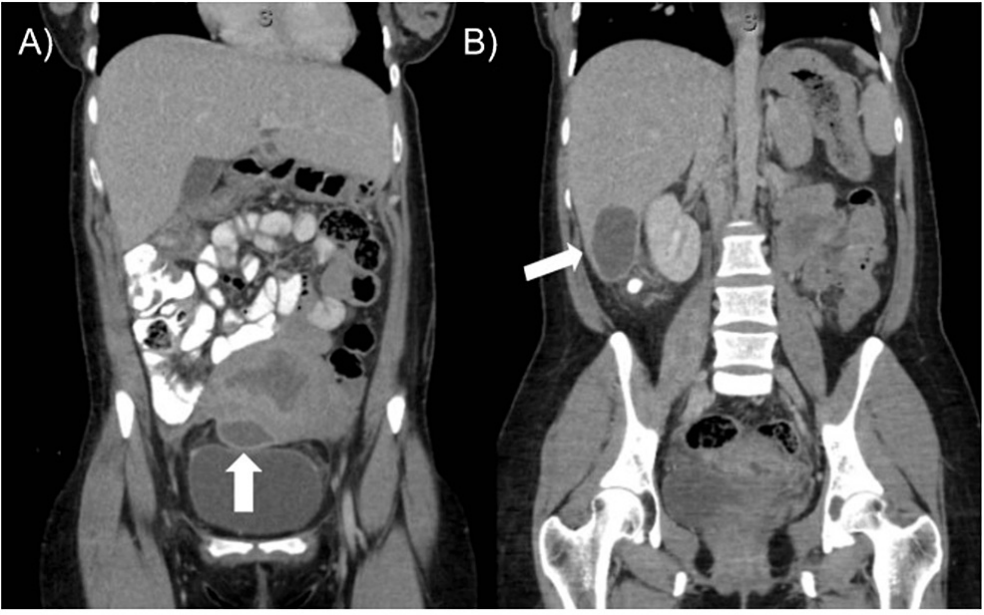
Computed tomography imaging reveals periuterine and subhepatic abscesses A) Coronal computed tomography (CT) scan from the patient’s presentation to the emergency room on postoperative day 12 showing an enlarged uterus measuring 14.3 x 10.3 x 7.1 cm with a 4.4 cm rim-enhancing, high-density cystic structure near the serosal surface of the anterior uterus. B) Coronal CT showing high-density 5.5 cm fluid collection on the inferior margin of the right lobe of the liver.

On presentation to the emergency department, the patient was febrile to 39.6°C. She denied chest pain, shortness of breath, nausea, vomiting, urinary symptoms, and vaginal pain or discharge. The physical exam was unremarkable. CBC demonstrated a WBC of 12.7 10^3^/μL and a platelet count of 753 10^3^/μL. Pelvic ultrasonography revealed no retained products of conception. Chest X-ray, urinalysis, and coronavirus disease 2019 (COVID-19) testing were negative. Blood cultures were drawn and the patient was admitted for IV antibiotics. A CT abdomen and pelvis (CTAP) was ordered and showed a 4.4 cm rim-enhancing fluid collection on the anterior wall of the uterus (Figure 1A). A 5.5 cm hyperdense cyst on the inferior margin of the right lobe of the liver was also present, deemed to likely be a cyst although there was no prior imaging for comparison (Figure 1B). There were no signs of thrombosis. Blood culture returned positive for *S. epidermidis*, deemed to be a likely contaminant. The patient was started on intravenous clindamycin 900 mg every eight hours and gentamicin 280 mg once daily. Hematology was consulted for the patient’s thrombocytosis, which was determined to be reactive, and she was recommended aspirin 325 mg daily until resolution.

**Figure 2:**
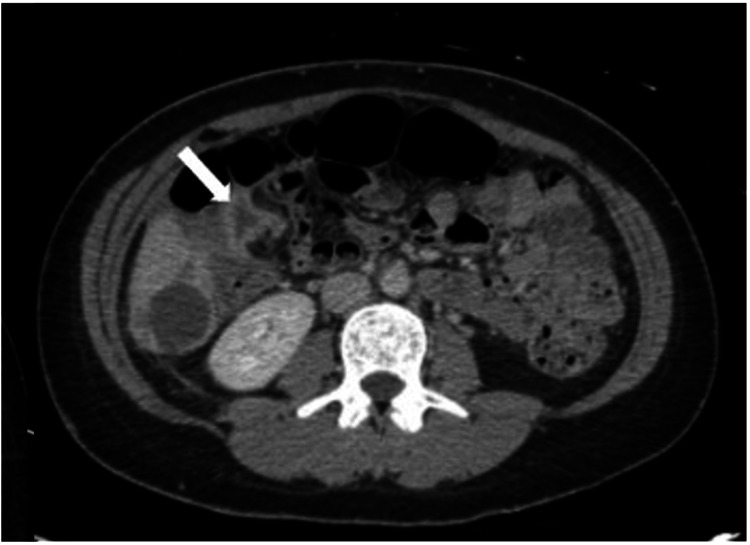
Computed tomography shows medial right pericolonic fluid collection Axial computed tomography (CT) from postoperative day 17 showing a 2.2 x 1.8 cm collapsed fluid collection involving the wall of the medial right colon.

Interventional radiology was consulted and performed percutaneous drainage of the periuterine fluid collection on postoperative day 15. Following the procedure, the patient became febrile to 40.3°C and required transfer to the medical ICU. Infectious disease was consulted, blood cultures were redrawn, and antibiotic coverage was broadened to intravenous vancomycin and cefepime. The following day, abscess cultures grew gram-positive anaerobes and meropenem was initiated in lieu of cefepime. The patient became febrile again in the evening. Repeat CTAP showed a decrease in the periuterine fluid pocket following drainage but an increase in the size of the right subcapsular hepatic fluid collection along with a potential pericolonic fluid collection (Figure 2). Radiology determined the liver process to be worrisome for abscess.

On postoperative day 18, the subhepatic fluid collection was drained percutaneously with an 8 French catheter that was left in situ, yielding 20 mL of purulent fluid. Uterine hematoma anaerobic culture grew 4+ *Peptoniphilus spp. *(no further speciation provided by the matrix-assisted laser desorption/ionization-time of flight (MALDI-TOF) analyzer) and the liver abscess anaerobic culture grew both 3+ *Peptoniphilus spp.* and 3+ *Finegoldia magna*. Antibiotics were then de-escalated to ertapenem 1 g daily. Repeat blood cultures were negative. By postoperative day 19, the drain had minimal output and was removed. The patient was then discharged home on ertapenem 1 g daily for four weeks followed by oral metronidazole 500 mg three times per day for two weeks for a total of six weeks of antibiotic therapy. The patient had a full recovery.

## Discussion

Differential diagnosis for late postpartum fever

The differential diagnosis for postpartum fever is broad, including surgical site infection, endometritis, mastitis or breast abscess, urinary tract infection, drug fever, and septic pelvic thrombophlebitis. This patient's clinical presentation of fever and thrombocytosis following intraamniotic infection raised suspicion for endometritis or septic pelvic thrombophlebitis. Urinalysis ruled out urinary tract infection and physical exam lowered suspicion for a surgical site infection. The patient’s continuing fevers despite broad-spectrum antibiotic coverage increased clinical suspicion for septic pelvic thrombophlebitis. However, CTAP showing a fluid collection on the anterior uterus suggested an infected hematoma. When the patient continued to be febrile despite IV antibiotics and percutaneous drainage, repeat CTAP pelvis demonstrated the previously thought liver cyst may actually be an abscess.

Discussion

*Peptoniphilus ssp.* and *Finegoldia magna* were formerly classified in the genus *Peptostreptococcus*, but more recent use of non-biochemical methods, including MALDI-TOF and 16s rDNA sequencing, has established both as unique anaerobic genera. As gram-positive anaerobic cocci (GPAC), these bacteria are commensals in human gastrointestinal and vaginal tracts [5]. Although these bacteria are often docile in immunocompetent hosts, they have been shown to engender unique pathologies in several case reports. In a population-based retrospective study by Badri et al. [6], several cases involving *Peptoniphilus* and *Finegoldia*-associated bacteremia, toxic shock syndrome, and infective endocarditis are described. Among the 226 episodes of GPAC-associated bacteremia identified, the 30-day mortality was found to be 11% [6]. In concert, a case series by Brown et al. [5] describes 15 cases of bacteremia associated with positive *Peptoniphilus ssp.* on blood cultures, including resulting pneumonia, urosepsis, septic abortion, pericarditis, and a pelvic abscess. The infections identified were often polymicrobial with a reported mortality of 20% (3 of 15). The remaining 12 were successfully treated with antibiotic therapy.

This present case is the first documentation of the authors’ knowledge of a *Peptoniphilus* or *Finegoldia*-associated hepatic abscess following a cesarean section. Literature on post-cesarean abscesses is scarce. One 2015 report from Muin et al. [7] introduces a case of a pelvic abscess two weeks after a cesarean section, but the recovered organisms were more common species: *Gardnerella vaginalis*, *Mycoplasma hominus*, and *Ureaplasma urealyticum*. The authors report the bacteria were initially resistant to systemic antibiotic therapy but the patient recovered after abscess drainage.

Similarly, the mode of transmission from the pelvis to the inferior right lobe of the liver is a highly unusual finding. Etiologies of liver abscesses can be classified as infectious, metastatic, or iatrogenic [8]. For infectious sources, the most common pathway is through the biliary tree (e.g. ascending cholangitis). Fifty percent of liver abscesses originate centrally in the right lobe due to its larger vascular supply [9], yet this bacterium seeded exceedingly uncommon foci in the peripheral aspect of the lower right lobe. Potential modalities of transmission include transportal circulation, direct inoculation, or hematogenous spread. However, uterine veins drain into the caval venous system, not the portal venous system. Direct inoculation is unlikely given that the abscess was not physically in contact with the hepatic surface; however, it cannot be ruled out, given that prolonged recumbency can contribute to the spread of pelvic abscesses. Although the patient’s blood cultures were negative upon readmission, it is quite possible that she experienced incidental bacteremia secondary to her acute chorioamnionitis at delivery, and the potential proteolytic properties of *Peptoniphilus spp.* and *Finegoldia magna* led to a rapidly growing intrahepatic abscess.

## Conclusions

This case highlights the importance of interdisciplinary communication in managing complex patient presentations. With varying symptomatology, hepatic abscesses can be difficult to diagnose clinically and are associated with significant mortality. A concerted effort between infectious disease specialists, obstetricians, and diagnostic and interventional radiologists proved integral in the diagnosis and treatment of this patient. The patient’s persistent fever refractory to broad antibiotic coverage led to suspicion of an additional unrecognized infection site, namely, the hepatic abscess that was first thought to be a benign hemangioma.

Although rare, given their high mortality, it is prudent to consider GPAC species, including *Peptoniphilus spp.* as a primary causative agent or as a polymicrobial contribution to postpartum fever. The authors hope this report will expand differential diagnoses for similar presentations and lead to a more expeditious diagnosis as well as provoke consideration of unique organisms of the reproductive tract as a potential culprit for unusual postoperative complications. 
